# Environmental and internal drivers of genotoxic carcinogens accumulation in botanicals and their preparations

**DOI:** 10.1007/s00204-025-04123-y

**Published:** 2025-07-11

**Authors:** Gerhard Prinsloo, Olusesan Ojo, Ivonne M. C. M. Rietjens

**Affiliations:** 1 Department of Agriculture and Animal Health, University of South Africa, Florida, South Africa; 2 Division of Toxicology, Wageningen University, Wageningen, The Netherlands

**Keywords:** Chemotypes, Cultivation methods, Genotoxic carcinogens, Herbal products, Medicinal plants, Phenology

## Abstract

The use of botanicals and herbal products is increasing globally, especially in developed countries, but their active ingredients are often poorly defined and inconsistently regulated. Moreover, the chemical composition of plants is usually influenced by internal factors (such as genetics, phenology, and age) and external influences (such as environmental conditions). These influences can significantly alter chemical profiles, which is critical for plants used in food, cosmetics, and medicine. Growing concern over harmful compounds in these products highlights the variability in their levels. Specifically, some compounds in plants are known to have genotoxic or carcinogenic effects, and their concentrations can vary greatly depending on these factors. This paper reviews how factors like water stress, soil nutrients, geographic location, and plant development affect the presence of hazardous substances in plants. While genetic factors like chemotypes and cultivars are well-studied, less is known about how environmental and developmental conditions affect chemically uniform plants. Overall, the current work emphasizes the unpredictability of these effects and underscores the need for further research to compare the levels of hazardous compounds in plants against established human safety thresholds, to better assess exposure risks in food, cosmetic and herbal products.

## Introduction

The global use of botanicals and herbal products is on the rise, particularly in developed countries. However, the active ingredients in these products are often not fully characterized or consistently regulated in terms of concentration (Indrayanto 2024; Heinrich 2015; Vargas-Murga et al. 2011; Shand et al. 2016; Barnes et al. 2016). Many consumers perceive botanicals as “natural” and therefore inherently “safe,” even though some plants can produce toxic compounds, including those that raise concerns because of their genotoxic and carcinogenic properties (Bardoloi and Soren 2022; Prinsloo et al. 2018; Götz et al. 2023). Meanwhile, cultivation is a way to ensure a sustainable supply of plant materials for use in plant-based supplements, herbal formulations, and traditional medicinal preparations. However, cultivation conditions significantly influence the chemical composition of plants, particularly when they alter the concentration levels of compounds of interest or concern (Aljaiyash et al. 2018; Pant et al. 2021; Vlachou et al. 2023). It is well known that the biosynthesis and accumulation of secondary plant products strongly depend on growth conditions, such as temperature, light, nutrients, water availability, the presence of microorganisms and other environmental effects that impact overall plant metabolism. These factors have been reported to influence the production of compounds of interest, such as secondary metabolites, both positively and negatively (Franz et al. 2011; Selmar and Kleinwächter 2013; Pant et al. 2021). This contrasts the common assumption that environmental stress conditions uniformly lead to an increase in all secondary metabolites in plants.

Environmental stress is often considered as a strategy to enhance the production or accumulation of compounds of interest. Plants exposed to drought stress frequently exhibit higher concentrations of secondary metabolites compared to well-watered counterparts (Al-Gabbiesh et al. 2015). However, the impact of environmental stress on secondary metabolites accumulation remains not fully understood, with studies reporting both positive and negative effects on the levels of constituents of interest (Al-Gabbiesh et al. 2015; Prinsloo and Nogemane 2018; Alhaithloul et al. 2019). For instance, water deficit has been shown to reduce the oil yield in rosemary (*Rosmarinus officinalis* L.) and anise (*Pimpinella anisum* L.) (Zehtab-Salmasi et al. 2001), while conversely increasing the concentration of phenolic capsaicinoids in peppers (*Capsicum annuum* L. var. *annuum*), thereby enhancing their pungency (Petropoulos et al. 2008). Similarly, both water supply and nitrogen fertilization influence alkaloid production in deadly nightshade (*Atropa belladonna*), with the highest yield of tropane alkaloids (hyoscyamine and scopolamine) occurring under optimal irrigation conditions. However, in other alkaloid-producing species, maximum alkaloid content is often achieved under conditions of severe water stress (Baricevic et al. 1999). Although water deficit may reduce overall plant biomass and oil yield per unit area (Al-Gabbiesh et al. 2015), it often leads to higher concentrations of compounds of interest. For instance, as observed in citronella grass (*Cymbopogon winterianus*), water stress significantly reduced growth and oil yield per area, but the oil yield based on fresh weight increased (Petropoulos et al. 2008). When cultivating economically valuable essential oil-producing plants that accumulate higher concentrations of desired compounds under water deficit conditions, closer plant spacing can help compensate for reduced biomass and increase oil yield per area (Petropoulos et al. 2008; Olle and Bender 2010). Similar strategies are suggested for other aromatic crops, as stress conditions tend to concentrate aroma-relevant compounds despite a decrease in overall biomass (Al-Gabbiesh et al. 2015).

In addition, internal factors can also affect the concentration levels of constituents of interest or concern. The occurrence of chemotypes with different concentration levels of constituents of interest or concern is quite common in, for instance, bitter and sweet fennel (*Foeniculum vulgare*) and various *Thymus* species. Thyme, specifically, includes nine subspecies and up to 13 chemotypes, based on the predominance of certain monoterpenoids in the essential oils (Satyal et al. 2016; van Wyk and Prinsloo 2020). Furthermore, the phenological stages of a plant species, such as optimal growth, flowering, fruiting, ripening, and seed production can influence the production of chemical compounds of interest. In addition, ontogeny, which refers to the physiologic changes an organism undergoes throughout its life (Pavarini et al. 2012), also impacts chemical compound production in plants. For example, in fennel, the main component, estragole, varied within the oils of the flower, unripe fruit, and ripe fruit (Ozcan et al. 2006; Franz et al. 2011). In *Cynoglossum officinale*, pyrrolizidine alkaloids (PAs) such as trachelanthamine, viridiflorine, rinderine, and heliosupine were most abundant in the inflorescences and young leaves (van Dam et al. 1995), whereas in various *Senecio* species, PAs like senecionine, integerrimine, and seneciphylline predominantly accumulated in the flower heads (Chizzola et al. 2019). Similarly, *Gynura bicolor* and *G. divaricata* exhibit variations in PA content and composition depending on the phenological stages and collection site. *G. divaricata* has the highest PA concentrations prior to flowering, while *G. bicolor* has the lowest PA concentrations at the same stage (Chen et al. 2017).

Although plant supplements and herbal formulations are generally considered “safe,” there are numerous reports highlighting the potential adverse effects of certain compounds found in plant materials. The presence and risk assessment of genotoxic carcinogens in plants used for food and medicine have garnered some attention (Prinsloo et al. 2018; Eisenreich et al. 2021; Götz et al. 2022). However, the quality assessment of food supplements and herbal formulations is limited, and the regulations surrounding the use of botanicals or botanical preparations containing known genotoxic carcinogens are often controversial and inconsistently enforced across different countries and jurisdictions (Heinrich 2015; Low et al. 2017). Since pre-market safety or quality assessments are not typically required for most botanicals or botanical preparations, the concentration and presence of harmful compounds are neither evaluated nor monitored. Therefore, this paper aims to provide an overview of the current information on the effects of external or environmental conditions, cultivation practices, as well as factors like plant species'chemotype, phenology, and ontogeny on the levels of genotoxic carcinogens in plants. This, no doubt, will offer a deeper understanding of the variability in concentrations and the key factors contributing to these fluctuations for future research.

## Methodology

### Search strategy and inclusion criteria

Searches were conducted on Google Scholar and ScienceDirect to identify publications addressing external factors that influence plant compounds, with a focus on those known to have adverse effects. Search terms included “environment”, “fertiliser”, “irrigation” or “cultivation”, with and without the inclusion of the terms “adverse effects”, “pyrrolizidine alkaloids”, “aristolochic acid” and “alkenylbenzenes”. Additional searches were conducted using key genotoxic carcinogens, including “safrole,” “estragole,” “methyleugenol,” “isoeugenol,” “myristicin,” “riddelliine,” “pulegone,” and “lasiocarpine” combined with terms like “cultivation,” “fertiliser,” “drought stress,” “irrigation,” and “post-harvest handling.” These searches aimed to identify studies exploring external factors that influence the chemical profiles and composition of plants, with a focus on genotoxic and carcinogenic compounds.

Furthermore, searches were conducted on Google Scholar and ScienceDirect using combinations of keywords such as “phylogeny,” “chemotypes,” “ontogeny,” “seasonal changes,” “concentration variations,” “plant age,” and “distribution,” along with the scientific names of the plant species of interest. Boolean operators were used to include terms like “chemical profiles,” “concentrations,” and “secondary metabolite production” to identify literature focused on the influence of internal factors on the production, content, and concentration of secondary metabolites. Similarly, Google searches were conducted to locate Monographs published by the International Agency for Research on Cancer (IARC), which classify various substances and botanicals based on their carcinogenic risk to humans. If a specific substance had not been evaluated by the IARC, further searches were carried out to obtain information from the European Chemicals Agency (ECHA).

### Exclusion criteria

Studies examining the impact of cultivation on medicinal plants were generally excluded if they did not quantify its effect on the chemical profile and composition—unless they offered novel insights into the plants’ chemical characteristics. Even though many papers report the effects of external and internal factors, including genetic factors on the chemical profiles of plants, only papers reporting the selected internal, as well as external effects on the concentrations of known genotoxic carcinogens were consulted. Plant genetic factors that influence secondary metabolite production in plants, including the production and content of genotoxic carcinogens, were also excluded.

## Results and discussion

Several plant species contain potentially genotoxic and carcinogenic chemicals. These include known carcinogens like pyrrolizidine alkaloids (PAs), as well as compounds with suspected genotoxic effects, such as the terpenes estragole (methyl chavicol) and *trans*-anethole. In addition, alkenylbenzenes such as *β*-asarone, pulegone, isoeugenol, methyleugenol, elemicin, apiole, and myristicin are also of concern. Many of these plants are used in the culinary industry or as ethnobotanical medicines, raising concerns about their safety (Table 1). A total of 96 peer-reviewed articles, theses, and books were reviewed in this study. Based on the reviewed literature, research often focuses on essential oils, with limited reporting on the content of these compounds in other parts of the plants. In addition, the essential oils studied are frequently not the same as those used in food or botanical preparations, emphasizing the need for studies that report accurate concentrations of potentially harmful compounds.

**Table 1:** A summary of some of the most important groups of genotoxic carcinogens, the plant families and plant species in which the substances were reported

Group of genotoxic carcinogens	Genotoxic carcinogen	Plant families	Plant species	References
Alkenylbenzenes	Methyl chavicol (estragole)	Lamiaceae Apiaceae	*Thymus vulgaris, Ocimum basilicum, Foeniculum vulgare, Pimpenella anisum, Petroselinum crispum*	Suparmi et al. (2018) Khalid (2006) Simon et al. (1992) Acimovic et al. (2015) Vokk et al. (2011)
	*β*-asarone Methyleugenol Isoeugenol	Araceae Apiaceae Lamiaceae Saururaceae	*Acorus calamus, Anthriscus cerefolium, Thymus vulgaris,* *Thymus daenensis, Laurus nobilis, Foeniculum vulgare,* *Anemopsis californica, Pimpenella anisum*	Kumar et al. (2016) El Gendy et al. (2015), Maatallah et al. (2016), Medina-Holguin et al. (2007) Pino et al. (1993) Suparmi et al. (2018)
	Myristicin	Apiaceae	*Foeniculum vulgare* *Anethum graveolens* *Petroselinum crispum*	Atta-Aly (2001) Vokk et al. (2011)
	Elemicin	Lauraceae Saururaceae	*Laurus nobilis* *Petroselinum crispum* *Anemopsis californica*	Marzouki et al. (2009) Maatallah et al. (2016) Medina-Holguin et al. (2007), Vokk et al. (2011)
	Apiole	Apiaceae	*Foeniculum vulgare* *Anethum graveolens* *Petroselinum crispum*	Bowes and Zheljazkov (2004) Hellal et al. (2011) Petropoulos et al. (2008)
Pyrrolizidine alkaloids	Lasiocarpine, senkirkine monocrotaline, riddelliine Senecionine, jacobine	Asteraceae	*Senecio spp., Vernonia amygdalina,* *Callilepis laureola, Gynura* spp. *Symphytum* spp.	El-Shazly (2002), Kirk et al. (2010), Letsyo et al. (2017) Chen et al. (2017) Dreger et al. (2009)
Monoterpene	t-anethole	Apiaceae	*Foeniculum vulgare, Pimpenella anisum*	Atta-Aly (2001), Bowes and Zheljazkov (2004), Acimovic et al. (2015)
Monoterpene ketone	Pulegone	Apiaceae Lamiaceae	*Anthriscus cerefolium* *Mentha* spp. *Ocimum basilicum* *Clinopodium nepeta*	Zheljazkov and Margina (1996), Pirbalouti et al. (2013), El Gendy et al. (2015), Vlachou et al. (2023)

Table 2 presents studies examining the influence of both external and selected internal factors on potentially genotoxic and carcinogenic compounds in essential oils. It is evident from the table that multiple cultivar or ecotypes of the same species were used in the studies. This suggests that while the applied treatments may lead to variations in the concentrations of these harmful substances, the role of genetic variability as a contributing factor cannot be excluded. Genetic variability was, however, not included as an internal factor in this review, as its effects are already well-known and extensively studied. From Table 2, it is also evident that some treatments caused significant changes of over 25% for certain components, while most of the treatments led to only minor changes of less than 2% in the substances measured. One of the key characteristics of carcinogens is that they are genotoxic (Smith et al. 2016). These key characteristics were also recently introduced into the IARC working groups’ evaluation process (IARC 2021), particularly with reference to mechanistic studies.

**Table 2:** Studies involving the effects of treatments on the percentages and fold changes of potential genotoxic carcinogenic compounds in essential oils

External/internal factors	Plant species	Treatment	Genotoxic carcinogen	% substance in essential oil before treatment	% substance in essential oil after treatment	% difference	Fold change	References
External	*Anethum graveolens*	N	apiole	Control 3.01	N1 18.43	+ 15.42	+ 5.12	Hellal et al. (2011)
	*A. graveolens*	N and biofertiliser	apiole	Control 3.01	N2 31.01	+ 28	+ 9.30	Hellal et al. (2011)
	*Petroselinum* spp. Plain-leaf parsley	45–60% Water deficit	myristicin	Control 28.63	33.35	+ 4.72	+ 0.16	Petropoulos et al. (2008)
	Curly-leaf parsley	45–60% Water deficit	myristicin	Control 61.09	41.20	− 19.89	− 0.32	Petropoulos et al. (2008)
	Turnip-rooted parsley	45–60% Water deficit	myristicin in roots	Control 13.55	7.7	− 5.85	− 0.43	Petropoulos et al. (2008)
	Plain-leaf parsley	45–60% Water deficit	apiole	Control 2.91	6.35	+ 3.44	+ 1.18	Petropoulos et al. (2008)
	Curly-leaf parsley	45–60% Water deficit	apiole	Control 2.96	2.95	− 0.01	+ 0.00	Petropoulos et al. (2008)
	Turnip-rooted parsley	45–60% Water deficit	apiole in roots	Control 23.59	25.7	+ 2.11	+ 0.09	Petropoulos et al. (2008)
	*Foeniculum vulgare* cv Dolce	Organic and inorganic N (ammonium nitrate)	myristicin	Organic 0.199	Inorganic 0.274	+ 0.075	+ 0.38	Atta-Aly (2001)
	*F. vulgare* cv Dolce	Organic and inorganic N (ammonium nitrate)	apiole	Organic 0.200	Inorganic 0.167	− 0.033	− 0.17	Atta-Aly (2001)
	*F. vulgare* cv Dolce	Organic and inorganic N (ammonium nitrate)	t-anethole	Organic 49.19	Inorganic 78.61	+ 29.42	+ 0.60	Atta-Aly (2001)
	*F. vulgare* cv Dolce	Organic and inorganic N (ammonium nitrate)	estragole (methyl chavicol)	Organic 2.316	Inorganic 3.151	+ 0.835	+ 0.36	Atta-Aly (2001)
	*F. vulgare* cv Azoricum	Organic and inorganic N (ammonium nitrate)	t-anethole	Organic 56.02	Inorganic 59.98	+ 3.96	+ 0.07	Atta-Aly (2001)
	*F. vulgare* cv Azoricum	Organic and inorganic N (ammonium nitrate)	estragole	Organic 1.795	Inorganic 3.247	+ 1.452	+ 0.81	Atta-Aly (2001)
	*Laurus nobilis* ecotype Annaba	Water stress	elimicin	Field capacity trace	20% FC trace	0		Maatallah et al. (2016)
	*L. nobilis* ecotype Bardo	Water stress	elimicin	Field capacity trace	20% FC trace	0		Maatallah et al. (2016)
	*L. nobilis* ecotype Annaba	Water stress	estragole	Field capacity 0.18	20% FC trace	− 0.18		Maatallah et al. (2016)
	*L. nobilis* ecotype Bardo	Water stress	estragole	Field capacity trace	20% FC trace	0		Maatallah et al. (2016)
	*L. nobilis* ecotype Annaba	Water stress	methyleugenol	Field capacity 6.0	20% FC trace	− 6.0		Maatallah et al. (2016)
	*L. nobilis* ecotype Bardo	Water stress	methyleugenol	Field capacity 1.0	20% FC 2.66	+ 1.66	+ 1.66	Maatallah et al. (2016)
	*L. nobilis* ecotype Annaba	Water stress	isoeugenol	Field capacity 0.4	20% FC 0.14	− 0.26	− 0.65	Maatallah et al. (2016)
	*L. nobilis* ecotype Bardo	Water stress	isoeugenol	Field capacity 0.44	20% FC 0.36	− 0.08	− 0.18	Maatallah et al. (2016)
	*Ocimum basilicum* cv purple landrace	Drying methods	estragole	Fresh 65.63	Oven drying at 60 °C 31.47	− 34.16	− 0.52	Pirbalouti et al. (2013)
	*Acorus calamus*	Drying method	*β*-asarone	Sun drying 67.2	Drying 40 °C for 60 h 53.9	− 13.3	− 0.20	Kumar et al. (2016)
	*Thymus daenensis*	Water stress and manure	methyleugenol	Water stress and manure 0.02	All others trace	− 0.02		Askary et al. (2018)
	*Anthriscus cerefolium*	N and K fertilizer	methyleugenol	Control 48.71	N0-K60 62.03	+ 13.32	+ 0.27	El Gendy et al. (2015)
	*A. cerefolium*	N and K fertilizer	pulegone	Control 0.26	N0-K120 6.24	+ 4.98	+ 19.1	El Gendy et al. (2015)
	*A. cerefolium*	N and K fertilizer	estragole	Control 19.5	N120-K120 26.43	+ 6.93	+ 0.36	El Gendy et al. (2015)
	*Mentha piperita* cv Zephir	Fertilizer	pulegone	Control 0.64	NPK 1.02	+ 0.38	+ 0.59	Zheljazkov and Margina 1996)
	*M. piperita* cv Mentolna-18	Fertilizer	pulegone	Control 0.40	NPK 0.28	− 0.12	− 0.3	Zheljazkov and Margina 1996)
Phenology	*A. graveolens*	Season	myristicin	Winter 28.15	Summer 1.67	− 26.48	− 0.94	Vokk et al. (2011)
	*A. graveolens*	Season	apiole	Winter 0.62	Summer 0.05	− 0.57	− 0.92	Vokk et al. (2011)
	*Petroselinum crispum*	Season	myristicin	Winter 30.67	Summer 42.65	+ 11.98	+ 0.39	Vokk et al. (2011)
	*P. crispum*	Season	apiole	Winter 1.76	Summer 0.11	− 1.65		Vokk et al. (2011)
	*P. crispum*	Season	elimicin	Winter 2.46	Summer 0.15	− 2.31		Vokk et al. (2011)
	*F. vulgare*	Season	t-anethole	Summer 53.95	Spring 44.61	− 9.34		Stefanini et al. (2006a)
	*L. nobilis*	Vegetative stages	estragole	Flowering 0.2	Seed production trace	− 0.2		Marzouki et al. (2009)
	*L. nobilis*	Vegetative stages	methyleugenol	Flowering 14.3	Seed production 15.8	+ 1.5		Marzouki et al. (2009)
Chemotypes/ecotypes	*F. vulgare* cv Berfena	Locality	t-anethole	Locality Canning 54.9	Locality Truro 63.4	+ 8.5		Bowes and Zheljazkov (2004)
	*F. vulgare* cv Shumen	Locality	t-anethole	Locality Canning 47.1	Locality Truro 72.1	+ 25		Bowes and Zheljazkov (2004)
	*F. vulgare* cv Sweet Fennel	Locality	t-anethole	Locality Canning 47.2	Locality Truro 75.7	+ 28.5		Bowes and Zheljazkov (2004)
	*F. vulgare* cv Berfena	Locality	estragole	Locality Canning 1.6	Locality Truro 0.2	− 1.4		Bowes and Zheljazkov (2004)
	*F. vulgare* cv Shumen	Locality	estragole	Locality Canning 2.0	Locality Truro 3.7	+ 1.7		Bowes and Zheljazkov (2004)
	*F. vulgare* cv Sweet Fennel	Locality	estragole	Locality Canning 1.8	Locality Truro 2.3	+ 0.5		Bowes and Zheljazkov (2004)
	*Anemopsis californica* (roots)	Locality/elevation	elimicin	San Pedro 2169 µg/g (highest value)	Faywood 0 µg/g (lowest value)	− 2169 µg/g (100% df)		Medina-Holguin et al. (2007)
	*Anemopsis californica* (roots)	Locality/elevation	methyleugenol	Mesilla 38 138 µg/g (highest value)	City of Rocks 9538 µg/g (lowest value)	28 600 µg/g (75% df)		Medina-Holguin et al. (2007)
	*L. nobilis*	Countries	methyleugenol	Spain 3.8	France 1.4	− 2.4		Pino et al. (1993)
Ontogeny	*F. vulgare* cv Sweet fennel	Maturity	t-anethole	Immature 85.81	Full Mature 87.85	+ 2.04		Telci et al. (2009)
	*F. vulgare* cv Sweet fennel	Maturity	estragole	Immature 5.00	Full Mature 5.16	+ 0.16		Telci et al. (2009)

### Potential human genotoxic carcinogens

**Fig. 1:**
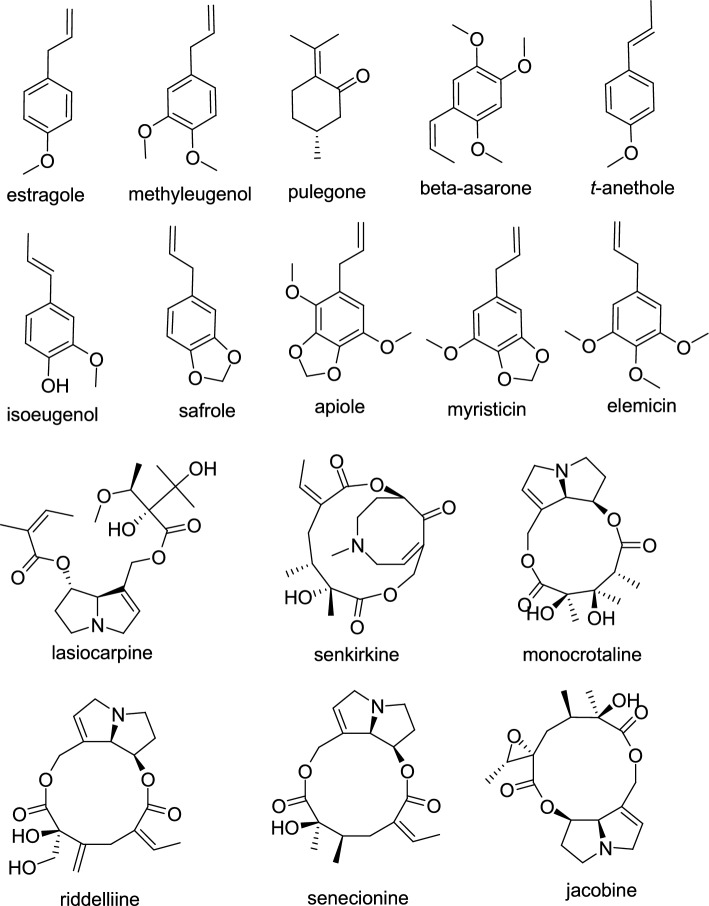
Chemical structures of some potential genotoxic carcinogens

Chemically, estragole is structurally similar to methyleugenol (Fig. 1), which the IARC has, in 2023, re-classified as “probably carcinogenic to humans” (Group 2 A) due to “sufficient” evidence of cancer in animal studies and “strong” mechanistic evidence from both humanized mice and studies in exposed humans (Rather et al. 2016; IARC 2013; Riboli et al. 2023). Although estragole has not been evaluated by the IARC, the European Chemical Agency (ECHA) noted estragole as “possibly carcinogenic to humans” (Group 2B) (https://echa.europa.eu/substance-information/-/substanceinfo/100.004.935, accessed April 7th, 2025). In a similar manner, the IARC working group assessed the compound pulegone for its potential carcinogenicity to humans and classified it as “possibly carcinogenic to humans” (Group 2B) (IARC 2016). Substances are typically classified as Group 2B when there is insufficient evidence regarding their carcinogenicity in humans, but there is enough evidence to suggest they are carcinogenic in experimental animals.

Again, the genotoxicity of *β-*asarone has been confirmed by Kevekordes et al. (2001) and due to the reported adverse effects, European regulatory authorities have prohibited or published recommendations on the use of *β-*asarone and preparations of *Acorus calamus* (Uebel et al. 2021). The genotoxicity of *t*-anethole was the focus of studies conducted by Newberne et al. (1989), Müller et al. (1994) and Gorelick (1995) but with inconsistent results. However, in a study by Auerbach et al. (2010), it was determined that *t-*anethole can be classified among the non-hepatocarcinogens.

The National Toxicology Program (2010) further reported the carcinogenic potential of isoeugenol, while Jeurissen (2007) examined the genotoxic carcinogenicity of safrole. In 2023, a Working Group of 25 scientists from 12 nations met at IARC in Lyon, France, to complete their assessment of the cancer-causing potential of certain compounds, of which isoeugenol was classified as"possibly carcinogenic to humans"(Group 2B) due to"sufficient"evidence of cancer observed in experimental animals (Riboli et al. 2023). In addition, Rietjens and Punt (2017) developed a model to enhance risk assessments for alkenylbenzenes, including apiole, myristicin, and elemicin, which are compounds that have not been evaluated by the IARC and for which evidence regarding their carcinogenicity in humans remains insufficient or inconsistent. However, there may be enough evidence of its carcinogenicity in animals, and according to the model proposed by Rietjens and Punt (2017), data from animal models can be more effectively extrapolated to the human context.

PAs are a group of toxic compounds and occur in plants such as *Cynoglossum* spp., *Crotalaria* spp., *Liparis* spp., *Ligularia* spp., and others. These alkaloids are also known as *Senecio* alkaloids, named for their high prevalence in *Senecio* species, which are frequently recognized as toxic plants (Baxter et al. 1998; Wink and Wyk 2008). Figure 1 depicts some PAs, including lasiocarpine, senkirkine monocrotaline, riddelliine, senecionine and jacobine, while Table 3 shows PA content variations in plant parts of wild and cultivated *Senecio* spp. and *Senecio* spp. adapted to local environments (ecotypes). Changes in chemical composition and concentrations in individual species, as well as changes in essential oil yield due to external and selected internal influences are outlined.

**Table 3:** Pyrrolizidine alkaloid content variations in plant parts of wild and cultivated *Senecio* spp. and *Senecio* spp. adapted to local environments (ecotypes)

Plant species	Plant parts	Genotoxic carcinogen	Wild plant Concentrations (µg/g)	Cultivated plant Concentration (µg/g)	Difference in µg/g and (percentage change)	References
*S. aegyptius* var. discoideus and *S. cineraria*	Flowers of wild and cultivated species	Total alkaloid	Wild *S. aegyptius* 4700	Cultivated *S. cineraria* 2300	− 2400 (51)	El-Shazly (2002)
*S. desfontainei* and *S. cineraria*	Flowers of wild and cultivated species	Total alkaloids	Wild *S. desfontainei* 6100	Cultivated *S. cineraria* 2300	− 3800 (63)	El-Shazly (2002)
*S. aegyptius* var. discoideus and *S. cineraria*	Roots of wild and cultivated species	Total alkaloids	Wild *S. aegyptius* 1000	Cultivated *S. cineraria* 1200	+ 200 (17)	El-Shazly (2002)
*S. desfontainei* and *S. cineraria*	Roots of wild and cultivated species	Total alkaloids	Wild *S. desfontainei* 1000	Cultivated *S. cineraria* 1200	+ 200 (17)	El-Shazly (2002)
*S. aegyptius* var. discoideus and *S. cineraria*	Leaves of wild and cultivated species	Total alkaloids	Wild *S. aegyptius* 2600	Cultivated *S. cineraria* 1200	− 1400 (54)	El-Shazly (2002)
*S. desfontainei* and *S. cineraria*	Leaves of wild and cultivated species	Total alkaloids	Wild *S. desfontainei* 1300	Cultivated *S. cineraria* 1200	− 100	El-Shazly (2002)
*Senecio jacobaea* and *Senecio aquaticus*	Adaptation of species to environment Roots	senecionine	*S. jacobea* 721	*S. aquaticus* 206	− 515 (71)	Kirk et al. (2010)
*Senecio jacobaea* and *Senecio aquaticus*	Adaptation of species to environment Shoots	senecionine	*S. jacobea* 185	*S. aquaticus* 185	– (–)	Kirk et al. (2010)
*Senecio jacobaea* and *Senecio aquaticus*	Adaptation of species to environment Shoots	seneciphylline	*S. jacobea* 261	*S. aquaticus* 326	+ 65 (20)	Kirk et al. (2010)
*Senecio jacobaea* and *Senecio aquaticus*	Adaptation of species to environment Roots	seneciphylline	*S. jacobea* 195	*S. aquaticus* 335	+ 140 (42)	Kirk et al. (2010)
*Senecio jacobaea* and *Senecio aquaticus*	Adaptation of species to environment Shoots	jacobine	*S. jacobea* 306	*S. aquaticus* 391	+ 85 (22)	Kirk et al. (2010)
*Senecio jacobaea* and *Senecio aquaticus*	Adaptation of species to environment Roots	jacobine	*S. jacobea* 89.6	*S. aquaticus* 0	− 89 (100)	Kirk et al. (2010)

### Botanical species with genotoxic carcinogens

From literature, some plant species have frequently been studied to assess how internal or external factors influence their chemical profiles, particularly in relation to genotoxic carcinogens. The main effects observed have been outlined below for each species individually.

#### *Petroselinum crispum* (Apiaceae)

Studies on *P. crispum* have shown that water deficit and seasonal changes influence the concentrations of the potentially genotoxic carcinogens myristicin and apiole (Petropoulos et al. 2008; Vokk et al. 2011). However, different parsley cultivars exhibited varied responses to water stress in terms of their myristicin and apiole levels (Table 2). For example, curly-leaf parsley showed a 19.89% decrease in myristicin (41.20%) as opposed to the control plants (61.09%), which is almost a two-fold decrease, whereas apiole content was hardly affected with the same treatment. On the contrary, myristicin content in plain-leaved parsley was only slightly affected with reduced water availability whereas apiole content increased more than twofold (Petropoulos et al. 2008). The effect of seasonal changes on the apiole and elemicin content in parsley showed that summer concentrations were lower than winter concentration for both compounds (Vokk et al. 2011). It was further found that harvesting frequency, season and fertilizer application influenced *Petroselinum* oil quality and yield (Petropoulos et al. 2008; Olle and Bender 2010). However, essential oil content can also change according to the hour of the day and the night (Olle and Bender 2010); therefore, the time plant material is harvested may also affect the content of compound of interest or concern. Frequent harvesting of parsley not only resulted in reduced biomass production at flowering stage but also a lower seed and oil yield (Osman and El-Wahab 2009; Olle and Bender 2010).

#### *Anethum graveolens* (Apiaceae)

The growth, biomass production, fruit yield and the essential oil composition of *A. graveolens* have shown to be affected by cultivation methods, harvesting frequency and season (Hellal et al. 2011). The apiole content, a major constituent of dill, increased following the application of chemical nitrogen fertilizer, with an even greater increase observed when chemical nitrogen fertilizer was combined with a biofertilizer (Table 2). The biofertilizer consisted of equal parts of the bacterial strains *Azotobacter chroococcum*, *Bacillus megatherium*, *Bacillus polymyxa*, *Azospirillum lipoferum, Pseudomonas fluorescens*, applied to the soil alongside the inorganic nitrogen fertilizer. These outcomes should be borne in mind when *A. graveolens* are cultivated and harvested for consumption, either as food or as ethnobotanical medicine. The addition of chemical nitrogen fertilizer and biofertilizer promotes plant growth and increases biomass, fruit yield and essential oil yield. However, it also increases the apiole content, which may lead to adverse effects in humans after long-term consumption. Dill harvesting throughout the growing season resulted in reduction in plant weight at flowering stage due to a reduction in the number of umbels, seed and oil yield per plant (Osman and El-Wahab 2009; Olle and Bender 2010). The essential oil yield of *A. graveolens* was slightly higher in summer when compared to winter, however seasons significantly affected the myristicin content in the oil with a decrease in content in summer as opposed to the winter content. The summer apiole content (0.05%) is lower than that of the winter content (Table 2; Vokk et al. 2011).

#### *Foeniculum vulgare* (Apiaceae)

*Foeniculum vulgare* is a plant species well-researched for its chemical content and essential oil yield, which vary depending on factors such as developmental stage, age, cultivar, season, and chemotype, as well as external conditions like soil nutrient levels, salinity, and water availability (Acimovic et al. 2015). Essential oil yield from *F. vulgare* differed depending on the plant part (leaves and stems, flower buds, inflorescences at anthesis, green seeds, ripe seeds, and dry seeds) as well as the season (Stefanini et al. 2006a). Drought stress increased the volatile oil yield (Mohamed and Abdu 2004) but it declined with fruit maturity (Telci et al. 2009; Olle and Bender 2010), with the highest oil yields found in plants harvested at waxy seed maturity stage in all the varieties tested (Marotti et al. 1993). Essential oil yield was increased with application of N, P, organic fertilizers and trace elements (Khalid 2006; Mohamed and Abdu 2004; Olle and Bender 2010), although salinity resulted in a progressive decline in oil content (Ashraf and Akhtar 2004) as well as the *t*-anethole content in oil (El-Wahab 2007; Olle and Bender 2010).

Again, the compounds *t*-anethole, apiole and estragole often differ between cultivars, locality, year of harvest as well as the seeding date. In a study by Bowes and Zheljazkov (2004), apiole was only present in one cultivar for all localities and years of harvest. However, *t-*anethole content in different *F. vulgare* cultivars varied widely for different localities, which can often be attributed to chemotypes (Table 2; Bowes and Zheljazkov 2004). In another study, the levels of *t*-anethole, myristicin, estragole, and apiole varied between two fennel cultivars subjected to organic and inorganic nitrogen (ammonium nitrate) treatments (Atta-Aly 2001). In *F. vulgare* cv. Dolce, myristicin, apiole, and estragole contents showed only slight differences between treatments: myristicin and estragole were slightly higher with inorganic nitrogen, while apiole was slightly higher with organic nitrogen (Table 2). In contrast, *t*-anethole content in this cultivar exhibited a nearly twofold increase under inorganic nitrogen treatment compared to organic nitrogen application (Atta-Aly 2001). In *F. vulgare* cv. Azoricum, the *t*-anethole content was slightly higher following inorganic nitrogen application compared to organic nitrogen application. However, estragole content was nearly twice as high with inorganic nitrogen than with organic nitrogen (Atta-Aly 2001). Seasonal fluctuations in *t*-anethole and estragole levels in *F. vulgare* have also been observed (Stefanini et al. 2006b). The *t-*anethole content in *F. vulgare* var. dulcis was 1.2-fold higher in summer than in winter while the estragole content showed only a slightly higher concentration in summer than in winter (Stefanini et al. 2006b).

#### *Mentha* spp. (Lamiaceae)

Investigation on the application of various combinations of nitrogen (N), phosphorus (P), and potassium (K) resulted in significant differences in oil yield and chemical composition of *Mentha piperita* and *Mentha arvensis* cultivars. However, fertilizer application did not consistently lead to higher oil yields compared to the unfertilized control samples; in some cases, the control samples even produced higher yields than the fertilized ones. As for chemical content, especially pulegone showed an increase in one cultivar with fertilizer application (Table 2), although other cultivars showed less pronounced differences between the various fertilizer applications (Zheljazkov and Margina 1996).

#### *Laurus nobilis* (Lauraceae)

*L. nobilis* is a very important medicinal and aromatic plant used in food and for therapeutic indications. Studies showed that drought stress, locality, ecotypes (subpopulations of a species which are adapted to their respective local external conditions) and seasonal influences, which affect a species’ phenological stages, affect the essential oil yield and chemical concentrations of *L. nobilis* leaves (Marzouki et al. 2009). Marzouki et al., (2009) found that the elemicin content did not differ between the flowering and the seed production stages. However, estragole was detected at a concentration of 0.2% of the total area during flowering but was only present in trace amounts during the seed production stage, indicating a decrease in estragole content after flowering. To the contrary, methyleugenol showed a small (1.10-fold) increase from the flowering stage to the seed production stage. Furthermore, geographic data revealed that locality affected the composition and presence of methyleugenol and isoeugenol (Pino et al. 1993), whereas ecotypes indicated varying concentrations of the genotoxic carcinogens methyleugenol and elimicin (Maatallah et al. 2016) (Table 2).

#### *Thymus* spp. (Lamiaceae)

Various *Thymus* species have been investigated to determine variations in essential oil composition and how it is affected by external factors. Suparmi et al. (2018) confirmed the presence of the potentially harmful compounds estragole and methyleugenol in *T. vulgaris*, although the effects of external and internal factors on these potentially genotoxic carcinogenic compounds are rarely recorded. Regarding essential oil yields, Askary et al. (2018) showed that drought stress and manure application affected the oil yield and composition of *T. daenensis* var. celak and *T. vulgaris* essential oils. When a 67% water deficit was applied, essential oil yield increased by 1.17 times compared to full irrigation at 100% field capacity. In contrast, a 33% water deficit resulted in a 1.31-fold decrease in essential oil yield relative to full irrigation. In addition, applying 30 tons per hectare of manure to thyme plants led to a 1.35-fold increase in essential oil yield compared to untreated plants (Askary et al. 2018). Other studies reported the effect of different fertilizer applications and post-harvest handling methods on chemical constituents of *T. daenensis*, *T. leptobotrys* and *T. maroccanus*, but only quantified the effects of these treatments on their major volatile components, for example carvole, but no complete compositional analysis for these species were reported (Alaoui Jamali et al. 2014; Dehghani Mashkani et al. 2018; Udagawa 1995). Methyleugenol was detected exclusively in *T. daenensis* under water stress conditions combined with manure application in the first year of study (2015). It was not found under water stress without manure in 2015, nor was it present in the following year (2016) under any conditions, with or without manure application (Table 2). The presence of estragole was not reported in any of the two *Thymus* species investigated by Askary et al., (2018).

#### *Anemopsis californica* (Saururaceae)

The Saururaceae consists of five genera but the genus *Anemopsis* is represented by only one species, *Anemopsis california.* Both the roots/rhizomes and aerial parts are used medicinally; however, the roots/rhizomes are more potent than the leaves. The minimum lethal dose (LD50) for the roots/rhizomes is 316 mg/kg, while the aerial parts showed no lethality even at doses up to 1 g/kg (Medina-Holguin et al. 2007). The essential oil of this species has been extensively studied, revealing that the oils extracted from its leaves and roots contain high percentages of methyleugenol and elemicin. In addition, *t*-anethole was also detected (Medina-Holguin et al. 2007; Medina-Holguín et al. 2008).

Three distinct chemotypes have been recorded for *A. californica*, with chemotypes being differentiated according to their methyleugenol, elimicin and thymol/piperitone content (Saville 2022). A concerning characteristic of *A. californica* is that they are known to readily absorb arsenic and other heavy metals from groundwater, which pose a serious health threat to users of ethnobotanical medicinal preparations made from the roots/rhizomes of this species (Saville 2022). Medina-Holguin et al., (2007) determined that there are connotations between chemical concentration variability and environmental conditions such as temperature and precipitation, as well as different altitudes (elevation). However, irrigation and fertilizer application only meekly affected the chemical content of *A. californica* root/rhizome essential oils.

#### *Anthriscus cerefolium* (Apiaceae)

*Anthriscus cerefolium* contains genotoxic carcinogens namely methyleugenol, methyl chavicol (estragole) and pulegone. The application of sodium (Na) and potassium (K) fertilizers increased the essential oil yield under water stressed conditions and consequently affected the concentrations of methyleugenol, estragole and pulegone with an increase in their contents in the oil (El Gendy et al. 2015). Chervil grown in different regions of Egypt exhibited differences in the composition of its essential oil, with estragole content ranging from 16.23% to 18.04% and methyleugenol content ranging from 43.81% to 47.25% across the four locations (Hendawy et al. 2019).

#### *Acorus calamus* (Araceae)

The aromatic rhizomes of *Acorus calamus* L. are widely used to alleviate stomach cramps, dysentery, and asthma. They also serve as anthelmintics, insecticides, tonics, and stimulants. The presence of *β*-asarone in *A. calamus* along with its genotoxic and carcinogenic potential has led to recommendations against the use of plants containing this compound. In addition, methyleugenol, another “probably carcinogenic to humans”, has also been identified in this plant (McGaw et al. 2002; Haupenthal et al. 2017; Woerdenbag et al. 2012). Drying methods and distillation time were proven to affect the *β*-asarone content. Sun drying resulted in the highest essential oil content, however *β*-asarone content was lower in oven-dried plant material than in sun-dried plant material (Table 2) whereas methyleugenol was highest with shade and oven drying (Kumar et al. 2016).

#### *Senecio* spp. (Asteraceae)

Many plant species contain PAs, some of which are constituents of “concern” because of their hepatotoxicity and strong potential as genotoxic carcinogens. PAs have been identified in numerous traditional herbal remedies derived from plant species, such as *Vernonia amygdalina* and *Callilepis laureola*. However, they are particularly well-known in species of the *Senecio* genus, which are especially rich in PAs. PAs were also detected in honey from bees that visited the flowers of PA-containing plants (Briske and Camp 1982; Popat et al. 2001; Kirk et al. 2010; Al-Gabbiesh et al. 2015; Bovee et al. 2015; Letsyo et al. 2017).

The *Senecio* genus (Asteraceae) is a genus consisting of more than 1100 species and the production of PAs is widespread (Kirk et al. 2010; Walter et al. 2020). Due to the presence of PAs, many *Senecio* spp. have been investigated for their response to external stimuli. Species such as *Senecio longilobus* and *Senecio jacobaea* have been reported to show increased alkaloid production under water deficit and nutrient stress (Briske and Camp 1982; Kirk et al. 2010; Al-Gabbiesh et al. 2015; Bovee et al. 2015). Several PAs, including senecionine, seneciphylline, jacobine and riddelliine were identified in Egyptian *Senecio aegyptius* var. discoideus, *Senecio desfontainei* and *Senecio cineraria* species (El-Shazly 2002). PA content variations also occur in various plant parts of wild and cultivated *Senecio* spp. and in *Senecio* spp. adapted to local environments (ecotypes) (Table 3). The presence of senecionine and retrorsine in Rooibos (*Aspalathus linearis)* tea samples have been attributed to contamination with a weed plant, *Senecio angustifolius*, growing in the area. However, *Senecio burchelli* also contains small amounts of senkirkine and senecionine and has been suggested as additional source of PA contamination in Rooibos as these weeds may be harvested together with Rooibos due to their close resemblance with Rooibos plants (Van Wyk et al. 2017).

#### *Pimpinella anisum* (Apiaceae)

Studies on *Pimpinella anisum* showed that its growth is influenced by fertilizer application, drought stress, and developmental stage. In anise, essential oil production was found to increase with the application of nitrogen, phosphorus, and trace elements, although the specific oil components were not analyzed (Khalid 2012). Water deficit affected the growth, yield and essential oil production, with reduced oil production during drought stress (Zehtab-Salmasi et al. 2001; Olle and Bender 2010) whereas harvesting stages resulted in different oil yields throughout the growing season with seeds harvested in waxy stage having a higher oil content than those harvested at earlier or later stages (Zehtab-Salmasi et al. 2001; Stefanini et al. 2006b; Ozel 2009). No reports on changes in chemicals with potential adverse effects according to phenology, ontogeny or external factors were found, however, one study showed variations in chemical content according to chemotype (Arslan et al. 2004). Anise plants contain several potentially hazardous compounds, with high concentrations of *t*-anethole reported (80.36–94.46%) (Table 4), with its isomer methyl chavicol (estragole), as well as isoeugenol and methyleugenol also present (Acimovic et al. 2015). The *t-*anethole content in all 14 chemotypes analyzed varied, however, methyl chavicol, isoeugenol and methyleugenol were only present in certain chemotypes while absent in others. There were also some variations in content in the chemotypes these chemicals occurred in (Table 4).

**Table 4:** Variations in *t*-anethole, methyl chavicol (estragole), isoeugenol and methyleugenol % in *Pimpinella anisum* essential oil samples collected from 14 different locations (Arslan et al. 2004)

Chemical compound	Location 1	2	3	4	5	6	7	8	9	10	11	12	13	14
*t-*anethole	89.76	89.48	89.71	92.23	92.94	94.46	89.50	84.70	80.36	91.51	92.80	93.39	90.50	94.03
estragole	2.61	2.42	0.41	0.65	0.47	2.67	2.40	0.39	–	0.53	0.08	0.08	0.07	0.44
methyleugenol	0.38	1.18	0.40	–	0.51	0.02	1.01	0.30	–	0.02	0.01	–	–	–
isoeugenol	–	0.17	0.07	0.13	0.06	0.08	–	0.19	–	0.18	tr	tr	0.01	0.09

(–) not detected or not present

Tr = trace amounts

#### *Ocimum* spp*.* (Lamiaceae)

A few studies have reported the effect of season, fertilizer application, cultivar or variety, drought stress and post-harvest handling on the chemical profile and essential oil composition of *Ocimum basilicum* and high concentration of potentially genotoxic carcinogens found in food products such as basil-based pesto (Ramezani et al. 2009; Forouzandeh et al. 2012; Al-Gabbiesh et al. 2015; Al-Malahmeh et al. 2017). Estragole occurred in only two basil-based pesto preparations while two other samples contained myristicin, and one sample contained both myristicin and apiole in addition to methyleugenol (Al-Malahmeh et al. 2017). Hussain et al., (2008) documented the seasonal variations of various volatile components in *O. basilicum* essential oil across all four seasons; however, the potentially genotoxic carcinogens, which are the focus of this review, were not detected in the analyzed samples.

In the study of Khalid (2006), seedlings of *Ocimum basilicum* and *Ocimum americanum* were subjected to varying levels of water stress at 125%, 100%, 75%, and 50% of field water capacity over 2 consecutive years. The estragole content in *O. basilicum* remained relatively stable across all treatments, ranging from 34.0 to 35.0 in both years of the study. In contrast, methyleugenol content showed notable variation, with the highest levels (3.9) observed under 75% water stress and the lowest levels (1.0) recorded at 50% water stress. In *O. americanum*, estragole content over both years were, 22.9, 20.7, 24.9, and 25.6 for 125, 100, 75 and 50% water stress treatments respectively. Methyleugenol content for both years were 0.63, 3.71, 0.90 and 1.00 for 125, 100, 75 and 50% water stress treatments respectively and isoeugenol content for 125, 100, 75 and 50% water stress treatments were 1,74, 3.11, 1.14 and 1.00 respectively for both years.

Basil essential oil yield is affected by the deliberate induction of drought stress and by fertilizer application but only with slight changes in yield. For instance, with a 80% of field capacity water deficit, the essential oil yield was slightly increased whereas a 60% water deficit caused a more slight increase in essential oil yield compared to that of the control plants (Forouzandeh et al. 2012). Fertilizer application led to a 1.13-fold increase in essential oil yield in basil plants with compost and a 1.11-fold increase with manure. In contrast, inorganic fertilizer did not enhance essential oil yield, although it did improve both dry plant yield (kg/ha) and average plant dry weight (g) compared to the control (Forouzandeh et al. 2012).

In a water stress study conducted by Khalid (2006), the highest essential oil yields in *O. basilicum* were recorded under a 50% water deficit in both years of the study. In the first year, this treatment produced a greater yield than the 100% field capacity application, while in the second year, the increase was smaller but still higher than the full treatment. However, the highest total essential oil yield (g plant^−1^) for both years were achieved at a 75% water deficit, with a 1.7-fold higher volume obtained in the first year and a 1.4-fold higher volume obtained in the second year (Khalid 2006). For *O. americanum,* optimal percentage essential oil yield varied between the first and second years of the study. In the first year, maximum percentage oil yield was achieved with a 50% water deficit, with a higher volume obtained compared to a 100% field capacity application, whereas in the second year, the maximum percentage oil yield was achieved when the plants were provided with excess water (125% of field water capacity water stress application), with a higher volume obtained compared to that of a 100% field water capacity application. However, total essential oil yield (g plant^−1^) for *O. americanum* for both years were achieved by 75% water stress application, with a higher volume obtained compared to a 100% field capacity water application in the first year and a higher volume obtained compared to a 100% field water capacity application in the second year (Table 5; Khalid 2006). Post-harvest handling of basil further affected the quality and yield of oil. The highest essential oil yields were obtained from plants exposed to shade-drying followed by freeze-drying, while an increase in drying temperature significantly decreased the essential oil content. The major compounds, including methyl chavicol (estragole), showed varied concentrations with the different drying methods applied (Pirbalouti et al. 2013).

**Table 5:** Effects of water stress treatments on essential oil yield of *Ocimum* spp. (Khalid 2006)

*Ocimum* sp.	Water stress treatments (%)	Essential oil %		Total essential oil yield (g plant^−1^)	
		First year	Second year	First year	Second year
*O. basilicum*	125	0.36	0.32	0.63	0.6
	100	0.24	0.29	1.04	1.20
	75	0.33	0.31	1.83	1.68
	50	0.38	0.35	1.10	0.97
*O. americanum*	125	0.29	0.27	0.68	0.65
	100	0.23	0.21	1.23	1.16
	75	0.25	0.22	1.72	1.61
	50	0.30	0.25	1.25	1.07

## Concluding remarks and future perspectives

Despite the increasing interest in plant secondary metabolites for human use, limited information exists regarding the influence of external or environmental factors on the chemical profiles of plants. Even fewer studies have addressed the role of internal factors, such as phenological stage, ontogeny, and the presence of distinct chemotypes or ecotypes, particularly in relation to the concentration of compounds of toxicological or regulatory concern. Given the broad diversity of plant species known to contain potentially hazardous constituents, including those widely consumed as food or used in traditional medicine, this knowledge gap represents a significant area of concern warranting further investigation.

When investigating the effects of a specific treatment on the production and concentration of target compounds particularly secondary metabolites, it is critical to minimize the influence of external or environmental variables. Factors such as air temperature, light intensity, humidity, soil nutrient content, and soil temperature must be maintained under constant conditions to ensure that observed changes can be attributed solely to the treatment under investigation. Studies conducted in open or uncontrolled environments, as observed in several cited works, are subject to considerable variability due to daily and seasonal fluctuations in environmental conditions including precipitation, temperature, humidity, wind, soil temperature, irradiance, and photoperiod. These fluctuations act as stressors that may independently or interactively alter the biosynthesis of secondary metabolites, thereby compromising the reliability and reproducibility of the results.

Soil-related variables such as water retention capacity, depth, microbial composition and activity, texture, structure, nutrient availability, pH, and temperature further complicate open-environment studies. Moreover, the physiologic responses of plants to these environmental factors are species-specific and depend on the tolerance thresholds of each species. Notably, plants may exhibit broad tolerance to certain factors and narrow tolerance to others, necessitating an understanding of the synergistic and antagonistic effects of multiple stressors.

For example, in a study where the effects of water deficit on the production and concentration of compounds of interest or concern is the focus, physiologic stress responses can include stomatal opening/closure, turgor pressure and evapotranspiration. Experiments which focus on the effects of a specific treatment on the production/content or composition of chemical compounds of interest or concern should therefore either be conducted under strictly controlled conditions or researchers should consider the combined effects of environmental stresses when conducting a study under field conditions.

Future research should also incorporate the established maximum safe dosage limits for humans, as defined by regulatory authorities, and systematically compare these thresholds with the concentrations of genotoxic carcinogens present in plants exposed to various external and internal stimuli. Such comparisons are essential for accurately assessing the potential risk of human exposure to these hazardous compounds when such plants are utilized as food ingredients or medicinal products.

## Data Availability

Not applicable.

## Abbreviations

ECHA: European Chemicals Agency
IARC: International Agency for Research on Cancer
PAs: Pyrrolizidine alkaloids
